# Efficacy of SLNB in early endometrial adenocarcinoma in China: a retrospective cohort study based on inverse probability of treatment weighting

**DOI:** 10.1186/s12885-026-15748-2

**Published:** 2026-02-25

**Authors:** Lingjuan Hu, Xiaofeng Chen, Xudong Hu, Jingyuan Luo, Huanmei Lin, Xiujun Zhu, Yanmei Sun, Jing Xiao

**Affiliations:** 1 The Second Clinical College of Guangzhou University of Chinese Medicine, Guangzhou, Guangdong China; 2 Department of Gynecology, The Second Affiliated Hospital of Guangzhou University of Chinese Medicine, Guangzhou, Guangdong China; 3 State Key Laboratory of Traditional Chinese Medicine Syndrome, The Second Affiliated Hospital of Guangzhou University of Chinese Medicine, 55 N, Neihuanxi Road, Guangzhou, Guangdong 510006 China; 4 School of Chinese Medicine, Hong Kong Baptist University, Hong Kong, China

**Keywords:** Endometrial Cancer, Laparoscopic Surgery, Sentinel Lymph Node Biopsy, Postoperative Complications, Chinese patients

## Abstract

**Background:**

Endometrial cancer significantly impacts women’s reproductive health and quality of life, with lymph node metastasis serving as a crucial prognostic factor. Given the low metastasis rate, not all early-stage patients require lymph node dissection. The NCCN upgraded Sentinel Lymph Node Biopsy (SLNB) technology to Class 2A evidence. However, the applicability of SLNB for Chinese patients remains uncertain. This study evaluates the efficacy and safety of SLNB in a Chinese population.

**Methods:**

A retrospective cohort study was conducted at Guangdong Provincial Hospital of Chinese Medicine from August 2019 to December 2022, involving 166 early-stage endometrial adenocarcinoma patients. Of these, 137 patients were assessed as moderate to high-differentiated endometrioid adenocarcinoma. Patients were divided into Sentinel Lymph Node Biopsy (SLNB, *N* = 43) and systematic lymph node dissection (SLND, *N* = 94) groups, with a median follow-up time of 39 (24, 55) months. Primary outcomes were postoperative disease progression-free survival and one-month complication rates, with secondary endpoints including surgical parameters and quality of life indicators.

**Result:**

During the follow-up(median follow-up time 39 months), 1 recurrence occurred in the SLNB group, with no recurrences in the SLND group. Sentinel Lymph Node Biopsy (SLNB) showed no statistically significant differences compared with traditional Sentinel Lymph Node Dissection (SLND) in oncological assessment, with no significant variations in postoperative pathological staging (β = 0.80, 95%CI: -1.29 to 2.90, *p* = 0.455), pathological grading (β = -0.115, 95%CI: -0.99 to 0.76, *p* = 0.800), and additional treatment evaluation (β = -0.214, 95%CI: -1.52 to 1.09, *p* = 0.749), with one recurrence in the SLNB group during follow-up. SLNB significantly improved surgical indicators: operation time reduced by 85.13 min (*p* < 0.001), blood loss decreased by 44.82 ml (*p* < 0.001), hospital stay shortened by 1.19 days (*p* = 0.030), with significantly lower postoperative day 1 pain scores. At 6 h post-surgery, both groups showed significant BADL decline (β = -21.53, 95%CI: -25.7 to -17.4, *p* < 0.001), with less functional impairment in the SLNB group and significantly shorter urinary catheter placement time (β = + 1.24, 95%CI: 0.90 to 1.59, *p* < 0.001). Complication analysis revealed significantly higher rates of lymphatic reflux obstruction (β = 18.22, 95%CI: 17.38 to 19.05, *p* < 0.001) and intestinal obstruction (β = 18.17, 95%CI: 16.16 to 20.17, *p* < 0.001) in the SLND group, suggesting a marked advantage of SLNB in complication management.

**Conclusion:**

For early-stage endometrial cancer patients with moderate to high differentiation and lesions confined to the inner half of the myometrium, SLNB demonstrates safety and efficacy as a minimally invasive alternative, significantly improving surgical outcomes and patient prognosis.

**Supplementary Information:**

The online version contains supplementary material available at 10.1186/s12885-026-15748-2.

## Introduction

Endometrial cancer(EC) is the sixth most common cancer in women [1], the incidence has ubiquitously increased worldwide [2], Surgery is the primary treatment for women with EC, Total hysterectomy with bilateral salpingo- oophorectomy (BSO) is standard of care and can be performed by an open or a minimally invasive approach [3], Lymph node metastasis is an important indicator for evaluating the prognosis of patients with endometrial cancer and guiding postoperative treatment. However, not all early-stage endometrial cancer patients without high-risk factors require lymph node dissection, as the metastasis rate is only 4% [4]. Furthermore, traditional lymph node dissection may prolong surgery time, increase surgical risks, and result in postoperative complications [5] use of NCCN's sentinel lymph node (SLN) mapping algorithm for the surgical staging of endometrial cancer has gained significant acceptance and is now commonly applied in many practices.

However, whether SLNB should be recommended in Chinese patients is still lack of supporting evidence from real-world data. Guangdong Provincial Hospital of Traditional Chinese Medicine has been utilizing SLNB technology for treating early-stage endometrial cancer patients since 2019, and a comprehensive clinical dataset has been established. This study aims to evaluate the efficacy and safety of SLNB in early endometrial adenocarcinoma patients who underwent laparoscopic total hysterectomy with bilateral salpingo-oophorectomy by comparing with systematic lymph node dissection (SLND).

## Methods

### Study design

A Retrospective Cohort Study Using Inverse Probability of Treatment Weighting (IPTW). The reporting of this study was followed The Strengthening the Reporting of Observational Studies in Epidemiology (STROBE) Statement (ref: https://www.equator-network.org/reporting-guidelines/strobe/), the checklist can be found in the supplementary file. This study was approved by the Institutional Review Board of Guangdong Provincial Hospital of Chinese Medicine (Ethics Committee of Guangdong Provincial Hospital of Chinese Medicine ZE2024-144–01). Given the retrospective nature of this study, the requirement for written informed consent was waived by the ethics committee, and all patient data were anonymized prior to analysis.

### Patients

The study included 166 patients diagnosed with endometrial adenocarcinoma who were admitted for surgical treatment at the Guangdong Provincial Hospital of Chinese Medicine from August 2019 to December 2022.

### Inclusion and exclusion criteria

Inclusion criteria: Patients must have a preoperative diagnosis of endometrioid adenocarcinoma with moderate to high histological differentiation (nuclear grade 1–2), with imaging showing lesions within the inner half of the myometrium (stage IA) without pelvic lymph node metastasis. They should have undergone laparoscopic total hysterectomy with bilateral salpingo-oophorectomy, along with either sentinel lymph node dissection (SLNB) or systematic lymph node dissection (SLND), with or without para-aortic lymph node dissection.

Exclusion Criteria: Patients with simultaneous pelvic and abdominal malignant tumors; a history of uterine, tubal, or ovarian resection for other conditions; a history of pelvic lymph node dissection for other reasons; a history of pelvic radiotherapy; incomplete medical records.

### Research method

#### Complication assessment

Employing the validated Clavien-Dindo classification system to systematically assess postoperative complications within the first 30 days, providing standardized grading from minor (Grade I-II) to severe complications (Grade III-V) (Supplementary Material 1).

#### Pain assessment

Pain intensity was assessed using the internationally validated Numerical Rating Scale (NRS, range 0–10). This assessment was embedded within the routine postoperative monitoring protocol and was systematically documented by attending nurses on postoperative day 1, day 3, and at discharge to monitor dynamic pain progression during the early postoperative period. (Supplementary Material 2).

#### Functional status assessment

Patient self-care ability was evaluated using the validated Basic Activities of Daily Living (BADL) scale. This assessment formed part of the standard perioperative care pathway and was conducted at admission (baseline), 6 h post-surgery (recovery phase assessment), and prior to discharge to comprehensively track functional status changes throughout the perioperative period. (Supplementary Material 3).

Both pain assessment and functional status evaluation were integral components of the standardized perioperative assessment protocol for all surgical oncology patients in this study. These assessments were incorporated into our institution's *mandatory electronic health record (EHR) documentation system* and were consistently administered by trained nursing staff at designated time points, ensuring data completeness, reliability, and traceability for retrospective analysis.

### Surgical procedures and techniques

#### SLNB group

Prepare a 1.25 mg/mL indocyanine green (ICG) solution by dissolving 25 mg of ICG powder in 20 mL of sterile water for intravenous injection. Inject 1–2 mL of the diluted ICG at 3 and 9 o’clock positions on the cervix, then wait for 15–20 min before surgery begins. After abdominal lavage, collect the lavage fluid. Perform electrocautery to ligate the distal ends of both fallopian tubes, open the lateral peritoneum, and examine the pelvic and para-aortic lymph nodes under laparoscopic fluorescence mode. Identify green-stained lymphatic vessels and nodes; those showing first-stage staining are considered sentinel lymph nodes (SLNs). A successful SLN mapping is achieved if at least one clearly visible green-stained lymph node is detected in each pelvic side. Remove the stained lymph nodes for histopathological evaluation, followed by total hysterectomy with bilateral salpingo-oophorectomy.

Based on the NCCN guidelines, if sentinel lymph node mapping fails or shows any suspicious or enlarged lymph nodes, concurrent ipsilateral lymph node dissection is recommended, followed by total hysterectomy with bilateral salpingo-oophorectomy. The removed lymph nodes undergo histopathological examination using pathologic staging techniques. Initially, routine hematoxylin and eosin (H&E) staining is performed. If metastasis is detected, further analysis is unnecessary. If no metastasis is found, consecutive tissue sections are prepared at 50-micron intervals, with two slices taken from each level, approximately 5 microns thick. These sections are subjected to standard H&E staining and immunohistochemical staining for cytokeratin AE1:AE3. If the sentinel lymph node is positive, a hysterectomy with bilateral salpingo-oophorectomy, pelvic lymphadenectomy, and para-aortic lymph node dissection is performed.

#### SLND Group

Patients in this group underwent laparoscopic total hysterectomy with bilateral salpingo-oophorectomy and systematic pelvic lymphadenectomy. Para-aortic lymphadenectomy up to the level of the inferior mesenteric artery (IMA) or the left renal vein was additionally performed based on the following intraoperative findings: (1) Deep myometrial invasion (≥ 50%); or (2) Palpably enlarged or suspicious retroperitoneal lymph nodes.

### Observation indicators

*The primary outcomes*: recurrence rate, one-month postoperative complication incidence, and Clavien-Dindo classification.

*Secondary outcomes*: medical costs, hospital stay, surgical parameters, and patient quality of life.

*Collected data*: The study collected comprehensive patient data including demographic characteristics, medical history, preoperative assessments, surgical details, postoperative pathological staging, treatment plans, hospitalization information, and complications, with quality of life evaluated through NRS (Numeric Rating Scale, NRS) pain scores, BADL(Basic Activities of Daily Living, BADL) assessments, and functional recovery indicators such as urinary catheter removal time and first flatus time.

### Follow-up methods

Follow-up was conducted through outpatient re-examination and by phone. Patients were followed up every 3–6 months for the first year post-surgery and annually thereafter. The follow-up encompassed general condition, occurrence of both short-term and long-term complications after surgery, as well as the long-term treatment outcomes. Abdominal ultrasound or MRI/CT scans were performed as needed. Follow-up endpoints are defined as patient relapse, death, or loss to follow-up, with follow-up scheduled until September 2025.

### Statistical analysis

During data preparation and endpoint definition, this study adhered to the principle for handling loss to follow-up or incomplete follow-up data: loss to follow-up was explicitly defined as the termination point of event observation (a censoring event). Specifically, the censoring time point for a patient was defined as the date of their last confirmed follow-up contact (e.g., the date of the last outpatient review or telephone follow-up where their status was clearly documented). This approach ensured the utilization of all available and valid observational information up to the point of loss to follow-up in the analysis.

To mitigate potential selection bias between groups, IPTW based on propensity scores was applied. Propensity scores were first estimated using a logistic regression model including clinically relevant baseline covariates that may affect surgical or oncologic outcomes: age, body mass index (BMI), history of pelvic surgery, history of hypertension, history of diabetes, preoperative pathological acquisition method (using hysteroscopy or not), preoperative tumor differentiation, and preoperative activities of daily living (BADL) score. Each individual was then weighted by the inverse probability of receiving the treatment actually received, and the average treatment effect (ATE) was estimated.

Categorical variables were summarized as counts and percentages, and continuous variables as mean ± standard deviation or median (interquartile range), as appropriate. The χ^2^ test, Fisher’s exact test, or Mann–Whitney U test were used for categorical variables, and the Student’s t-test was used for continuous variables. Logistic regression analyses were performed to identify independent risk factors for postoperative complications.

All statistical analyses were conducted using SPSS version 26.0 (IBM Corp., Armonk, NY, USA) and R software (version 4.1.0). Two-tailed *P* values < 0.05 were considered statistically significant.

## Results

### Clinicopathologic characteristics

**Fig. 1:**
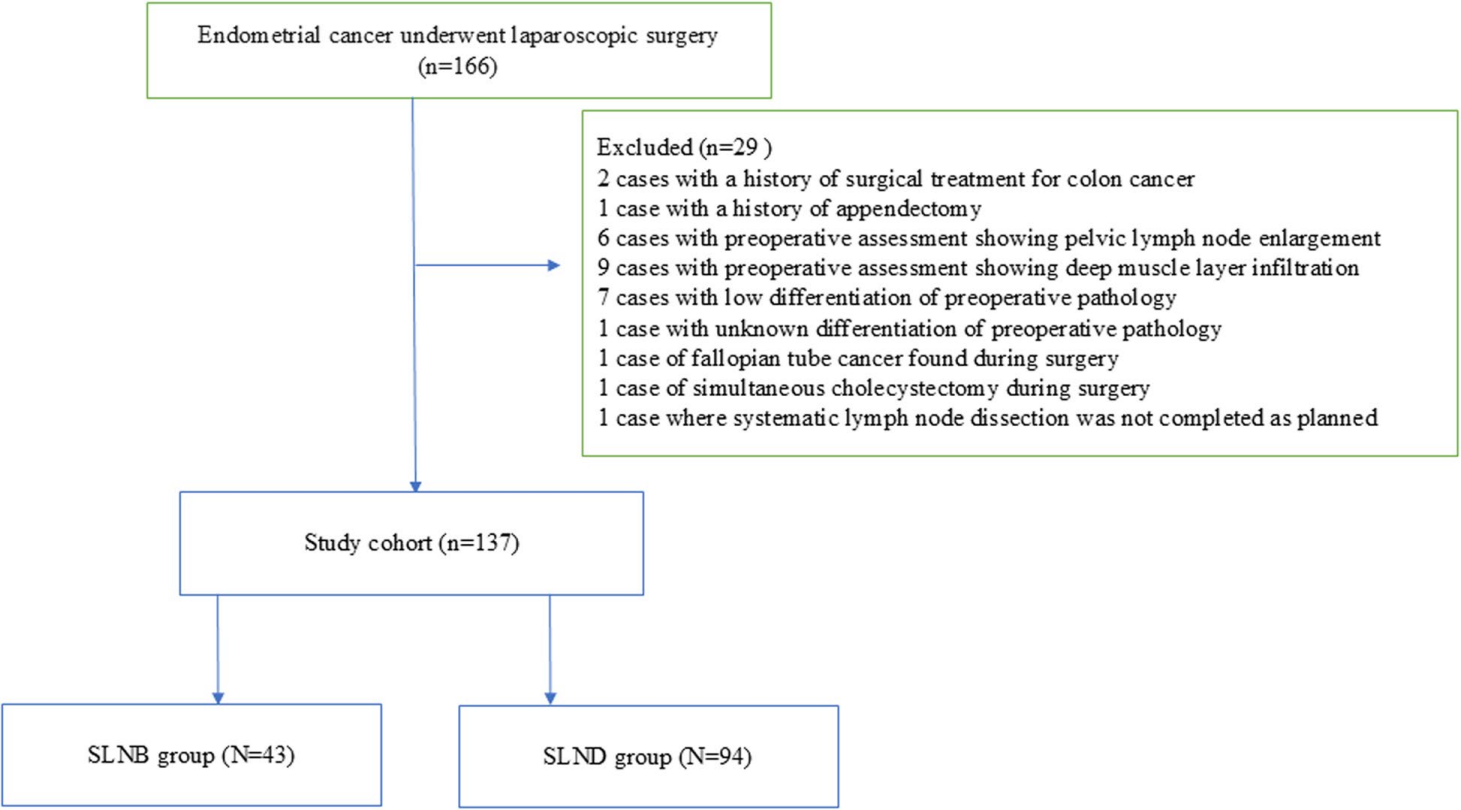
Case screening form

From 166 endometrial cancer patients undergoing laparoscopic surgery, the study rigorously screened and ultimately included 137 patients, divided into SLNB (*n* = 43) and SLND (*n* = 94) groups. The 29 excluded patients had reasons such as prior surgical history, preoperative imaging abnormalities, and unclear pathological characteristics (Fig. 1). Although no statistically significant differences were observed between groups in baseline clinicopathologic characteristics including age, body mass index, comorbidities, and preoperative pathological features, the researchers employed inverse probability of treatment weighting (IPTW) to minimize potential bias and ensure the reliability of the study results.The baseline clinicopathologic characteristics of patients in the SLNB and SLND groups are summarized in Table 1. Variables included age, body mass index (BMI), history of pelvic surgery, hypertension, diabetes, preoperative pathological acquisition method (using hysteroscopy or not), preoperative pathological differentiation, and preoperative activities of daily living (BADL) score. Although no statistically significant differences were observed between groups in these baseline variables (*P* > 0.05), inverse probability of treatment weighting (IPTW) was further applied to minimize potential confounding and improve the robustness of the comparisons (Table 1).

**Table 1:** Baseline clinicopathologic characteristics of patients in the SLNB and SLND groups before IPTW adjustment

Characteristicse	All patients	Group		*p*-value
		SLNB (*N* = 43)	SLND (*N* = 107)	
Age, x̅ ± s	51 (44, 55.5)	53 (48.25, 57)	51 (44, 55.5)	0.17
BMI, x̅ ± s	16.72 ± 2.53	15.92 ± 2.55	16.72 ± 2.53	0.093
Pelvic surgery history, n (%)				0.502
No	32 (74)	63 (67)	32 (74)	
Yes	11 (26)	31 (33)	11 (26)	
History of hypertension, n (%)				1
No	32 (74)	71 (76)	32 (74)	
Yes	11 (26)	23 (24)	11 (26)	
History of diabetes, n (%)				0.72
No	41 (95)	87 (93)	41 (95)	
Yes	2 (5)	7 (7)	2 (5)	
Preoperative pathological acquisition method (using hysteroscopy, n %)				0.754
No	3 (7)	10 (11)	3 (7)	
Yes	40 (93)	84 (89)	40 (93)	
Preoperative Pathological Stage Assessment	137 (100)	43 (100)	94 (100)	1
Preoperative Pathological Grade, n (%)				0.192
G1	93 (68)	33 (77)	60 (64)	
G2	44 (32)	10 (23)	34 (36)	
Preoperative Basic Activities of Daily Living (BADL) Scale Score				1
60	1 (1)	0 (0)	1 (1)	
75	1 (1)	0 (0)	1 (1)	
90	2 (1)	0 (0)	2 (2)	
95	2 (1)	0 (0)	2 (2)	
100	131 (96)	43 (100)	88 (94)	

*The difference was statistically significant (*P*< 0.05)

### Sentinel lymph node biopsy detection results

In a sentinel lymph node detection study involving 43 patients, successful lymph node visualization and detection was achieved in 40 cases (93%), with 2 cases (5%) of visualization failure and 1 case (2%) of successful visualization but undetected lymph nodes; the bilateral detection rate was 67% (29/43), with a total of 220 sentinel lymph nodes identified, predominantly distributed in the external iliac region (143, 65%), obturator fossa (44, 20%), internal iliac region (14, 6.4%), para-aortic area (12, 5.4%), common iliac artery region (1, 0.5%), and presacral area (6, 2.7%), demonstrating the high efficiency and extensive localization capabilities of SLNB in endometrial cancer lymph node detection (Table 2).

**Table 2:** Sentinel lymph node detection status

	*N* = 43	%
Successful lymph node visualization and detection cases	40	93
Failed visualization cases	2	5
Successful visualization but undetected lymph node cases	1	2
Bilateral detection rate	29	67
Sentinel lymph node localization		
Total	220	
External iliac area	143	65
Obturator fossa area	44	20
Internal iliac area	14	6.4
Para-aortic area	12	5.4
Common Iliac Artery area	1	0.5
Presacral area	6	2.7

### Lymph node yield in patients undergoing systematic lymphadenectomy

Systematic pelvic lymphadenectomy was performed in 94 patients on the right side and in 93 patients on the left (with one omission due to comorbidities), yielding a median of 12.0 nodes bilaterally. In contrast, para-aortic lymphadenectomy was not routinely performed. Consequently, the median para-aortic node count was 0.0 for the entire cohort (*N* = 94), as the procedure was selectively pursued in only 29 patients who presented intraoperative high-risk features. In this subgroup, the median node yield was 6.0 (Table 3).

**Table 3:** Lymph node yield in patients undergoing systematic lymphadenectomy

Lymph Node Basin	N	Min	Max	Median (IQR)	(Mean ± SD)	Total
Left Pelvic Lymph Nodes	93	4	26	12.0 (9.0–15.0)	(12.09 ± 4.45)	1124
Right Pelvic Lymph Nodes	94	3	29	12.0 (9.0–15.0)	(12.10 ± 4.91)	1137
Para-aortic Lymph Nodes	94	0	26	0.0 (0.0–4.0)	(2.33 ± 4.48)	219
*(Subgroup with dissection)*	*29*	*2*	*26*	*6.0 (4.0–10.0)*	*(7.55* ± *5.07)*	*219*

Para-aortic lymphadenectomy was not performed routinely. The main row for para-aortic nodes describes the yield in the entire cohort (*N* = 94), where a median of 0 indicates most patients did not undergo the procedure. The italicized subgroup row below details the yield specifically in the 29 patients who met intraoperative high-risk criteria and subsequently underwent para-aortic lymphadenectomy. One patient did not undergo left pelvic lymphadenectomy due to comorbidities

### Perioperative outcomes after IPTW adjustment

After inverse probability of treatment weighting, the perioperative outcomes between the SLNB and SLND groups were compared (Table 4). The operation time and intraoperative blood loss were significantly higher in the SLND group compared to the SLNB group (β = + 85.13, 95% CI: 59.9–110.4, *P* < 0.001; β = + 44.82, 95% CI: 28.4–61.2, *P* < 0.001). No significant differences were observed between the two groups in postoperative pathological stage, pathological grade, or the need for adjuvant therapy (*P* > 0.05).

**Table 4:** Perioperative outcomes of patients after IPTW adjustment

Observation Indicators	β (SLND vs SLNB)	95% CI	p-value
Operative Duration	85.13	(59.9, 110.4)	< 0.001*
Intraoperative Blood Loss	44.82	(28.4, 61.2)	< 0.001*
Postoperative Pathological Stage	0.8	(− 1.29, 2.90)	0.455
Postoperative Pathological Grade	− 0.115	(− 0.99, 0.76)	0.800
Need for Adjuvant Therapy	− 0.214	(− 1.52, 1.09)	0.749
Occurrence of Postoperative Complications	3.98	(0.74, 21.5)	0.105
Lymphatic reflux obstruction	18.22	(17.38, 19.05)	< 0.001*
Postoperative infection	1.955	(− 0.118, 4.028)	0.065
Postoperative intestinal obstruction	18.17	(16.16, 20.17)	< 0.001*
Postoperative thrombosis)	0.114	(− 2.21, 2.44)	0.923

^*^The difference was statistically significant (*P* < 0.05)

The SLND group (*n* = 94) showed a higher postoperative complication rate (24.5%) compared to the SLNB group (*n* = 43, 4.6%). However, the overall postoperative complication rate did not show statistically significant differences between the groups (β = 3.98, 95% CI: 0.74–21.5, *P* = 0.105). Notably, the SLND group exhibited significantly higher rates of specific postoperative events, such as lymphatic reflux obstruction (β = 18.22, 95% CI: 17.38–19.05, *P* < 0.001) and intestinal obstruction (β = 18.17, 95% CI: 16.16–20.17, *P* < 0.001) compared to the SLNB group. Postoperative infection (β = 1.955, *P* = 0.065) and thrombosis (β = 0.114, P = 0.923) rates showed no significant differences (Tables 4 and 5).

**Table 5:** Detailed postoperative complications and Clavien-Dindo classification

	SLNB (n,%)	SLND (n,%)	Clavien-Dindo
Total Patients	43	94	
Patients with Complications	2(4.6%)	23(24.5%)	
Complication Type Distribution			
Single Complication	2	20	
Multiple Complications	0	3*	
Lymphatic reflux obstruction	0	13	II
Postoperative infection	0	7	II
Postoperative intestinal obstruction	0	1	II
Postoperative thrombosis	0	4	II
Total	2	26	

^*^One patient had lymphocele with thrombosis, and two patients had lymphocele with infection

### Postoperative recovery and hospitalization evaluation after IPTW adjustment

The postoperative recovery indicators after IPTW adjustment are summarized in Table 6. The SLND group reported significantly higher postoperative pain scores on postoperative day 1 (β = + 0.92, 95% CI: 0.61–1.22, *P* < 0.001) and lower activities of daily living (BADL) scores at 6 h postoperatively (β = − 21.53, 95% CI: − 25.7 to − 17.4, *P* < 0.001) compared with the SLNB group. The BADL scores on the day of discharge showed no significant difference between groups (*P* = 0.143).

**Table 6:** Postoperative recovery and hospitalization outcomes after IPTW adjustment

Observation Indicators	β (SLND vs SLNB)	95% CI	*p*-value
Postoperative Day 1 NRS	+ 0.92	(0.61, 1.22)	< 0.001*
BADL at 6 Hours Postoperatively	− 21.53	(− 25.7, − 17.4)	< 0.001*
BADL at Discharge Day	− 1.70	(− 3.97, 0.56)	0.143
Urinary Catheter Removal Time	+ 1.24	(0.90, 1.59)	< 0.001*
Time to First Flatus	+ 0.35	(− 0.01, 0.70)	0.058
Total Hospital Stay	+ 1.04	(− 1.00, 3.08)	0.319
Postoperative Hospital Stay	+ 1.19	(0.12, 2.26)	0.030*
Hospitalization Cost	+ 5,197	(− 1,242, 11,636)	0.115

^*^The difference was statistically significant (*P* < 0.05)

The urinary catheter removal time was significantly longer in the SLND group (β = + 1.24, 95% CI: 0.90–1.59, *P* < 0.001), while the time to first flatus showed a trend toward delay but did not reach statistical significance (β = + 0.35, 95% CI: − 0.01 to 0.70, *P* = 0.058).

No significant difference was found in total hospitalization duration (β = + 1.04, 95% CI: − 1.00 to 3.08, *P* = 0.319), but postoperative hospitalization duration was slightly longer in the SLND group (β = + 1.19, 95% CI: 0.12–2.26, *P* = 0.030). Although the total hospitalization cost tended to be higher in the SLND group, the difference did not reach statistical significance (β = + 5197, 95% CI: − 1242 to 11,636, *P* = 0.115) (Table 6).

### Follow-up and recurrence situations

The median postoperative follow-up time was 39 months (interquartile range, 24–55 months) for all patients, 36 months (20.5–51.5) in the SLNB group, and 40 months (25.25–58.75) in the SLND group, with no significant difference between the two groups (*P* = 0.26). Only one recurrence occurred in the SLNB group, while no recurrence was observed in the SLND group (*P* = 0.314) (Table 7). Given the low number of recurrence events, no weighted analysis was performed. These findings suggest similar short-term oncologic safety between sentinel lymph node biopsy and systematic lymph node dissection in patients with early endometrial adenocarcinoma.

**Table 7:** Comparison of postoperative recurrence between two groups

Observation Indicators	Group		Total	p-value
	SLNB(n,%)	SLND(n,%)		
Postoperative follow-up duration (months), Median (Q1,Q3)	36 (20.5, 51.5)	40 (25.25, 58.75)	39 (24, 55)	0.26
Postoperative recurrence				0.31
NO	42(98)	94(100)		
Yes	1(2)	0(0)		

^*^The difference was statistically significant (*P* < 0.05)

## Discussion

While randomized trials have established the safety of SLNB in Western populations, data for Chinese patients remain limited; our study bridges this gap through real-world evidence. Although the sample size is limited, constraining the cohort scale, it reflects the early institutional adoption of SLNB technology in China and provides valuable real-world insights. By conducting a detailed analysis of SLNB performance in our Chinese cohort, we offer meaningful preliminary evidence for the application of this minimally invasive surgical technique in local medical practice.

This propensity-weighted, real-world cohort study using inverse probability of treatment weighting (IPTW) provides further contemporary evidence that sentinel lymph node biopsy (SLNB) is an accurate and less morbid alternative to complete lymphadenectomy (SLND) for selected early-stage endometrial cancer patients [6–8]. Our overall per-patient SLN detection rate was 93%, with bilateral mapping in 67% of patients and 220 sentinel nodes identified—predominantly in the external iliac and obturator basins—consistent with recent multicenter series and systematic reviews supporting high detection and negative predictive value when cervical injection of indocyanine green (ICG) and SLN algorithms are applied [9–11].

Oncologic outcomes in this cohort were comparable between SLNB and SLND: there were no statistically significant differences in postoperative FIGO pathological stage, tumor grade, or adjuvant treatment recommendations, and only one recurrence occurred in the SLNB group during follow-up [12, 13]. These findings align with emerging national and multicenter analyses showing no detriment in recurrence-free or cancer-specific survival with SLNB-based staging in appropriate early-stage populations when mapping algorithms and ultrastaging are used [13, 14].

The anatomic distribution of identified sentinel nodes in our study—external iliac (65%), obturator (20%), internal iliac (6.4%), para-aortic (5.4%) and presacral/common iliac rarities—mirrors known uterine drainage pathways and underlines the clinical importance of systematic intraoperative inspection for atypical drainage pathways, particularly para-aortic nodes in higher-risk patients [15, 16]. The observed para-aortic involvement (12 of 220 nodes, 5.4%) reinforces recommendations to consider side-specific or para-aortic assessment in patients with risk features for isolated para-aortic metastasis [16, 17].

Clinically meaningful perioperative benefits were evident for SLNB: operation time decreased by ~ 85 min, blood loss fell by ~ 45 mL, hospital stay shortened by ~ 1.2 days, and early postoperative pain and functional impairment were reduced [18–20]. Shorter urinary catheter duration and earlier recovery of activities of daily living were also noted. These favorable surgical metrics echo growing literature that links omission of routine lymphadenectomy to reduced operative morbidity, shorter recovery, and lower lymphatic complication rates (lymphedema, lymphocyst formation) [19–21].

Complications in the SLND group—particularly lymphatic reflux obstruction and intestinal obstruction—were observed at higher rates, consistent with meta-analyses documenting increased lymphatic morbidity after systematic lymphadenectomy [22, 23]. Such differences have important implications for postoperative quality of life and long-term functional outcomes and argue for SLNB adoption where oncologically appropriate [23, 24].

### Limitations

This is a single-country, single-center real-world study with a modest sample size and limited follow-up for long-term survival endpoints [25]. Although IPTW reduced measured confounding, residual unmeasured confounding (e.g., surgeon experience, tracer technique variations, BMI distribution) may remain and may explain the lower bilateral detection rate compared with some high-volume centers [25, 26]. We therefore urge cautious interpretation of survival inferences and recommend replication in larger, multicenter prospective cohorts [26, 27].

### Clinical implications and future directions

1. Standardize ICG injection technique and intraoperative mapping protocols, and institute structured training programs to reduce variability and improve bilateral mapping rates [28].
2. Rigorously apply SLN algorithms that mandate side-specific dissection for unmapped hemipelves and incorporate ultrastaging to detect low-volume metastatic disease [29].
3. Prospectively assess recurrence patterns, survival outcomes, and patient-reported quality of life in larger, ideally randomized or registry-based studies, and integrate molecular classification to refine individualized nodal management [13, 30].

## Conclusion

In early-stage endometrial cancer patients with lesions confined to the inner half of the myometrium and moderate-to-high differentiation, SLNB using ICG and a validated algorithm achieved high detection rates and oncologic comparability to SLND while reducing perioperative morbidity. Our results support expanded use of SLNB in appropriately selected Chinese patients within centers that provide standardized mapping and ultrastaging. Further confirmation of long-term oncologic safety in larger prospective studies is warranted.

## Supplementary Information

Supplementary Material 1

Supplementary Material 2

Supplementary Material 3

## Data Availability

The datasets generated and/or analyzed during the current study are not publicly available due to patient privacy and confidentiality restrictions, as mandated by the ethics committee that approved this research. However, the de-identified datasets are available from the corresponding author upon reasonable request. Data requests will be reviewed to ensure they align with the ethical approval and intended use of the data. Interested researchers should submit a formal request outlining their proposed analysis to the corresponding author.

## Abbreviations

NCCN: National Comprehensive Cancer Network
EC: Endometrial Cancer
SLN: Sentinel Lymph Node
SLNB: Sentinel Lymph Node Biopsy
SLND: Systematic Lymph Node Dissection
BADL: Basic Activities of Daily Living
NRS: Numerical Rating Scale
BSO: Bilateral Salpingo-Oophorectomy)
HER: Electronic Health Record
ICG: Indocyanine Green
IPTW: Inverse Probability of Treatment Weighting
MRI: Magnetic Resonance Imaging
CT: Computed Tomography
ATE: Average Treatment Effect
