# Supplementary material for: Efficacy of SLNB in early endometrial adenocarcinoma in China: a retrospective cohort study based on inverse probability of treatment weighting
Source: BMC Cancer. 2026 Feb 25;26:425. doi: 10.1186/s12885-026-15748-2 (PMC13041019; doi:10.1186/s12885-026-15748-2)
Supplement: Supplementary file 1 — Supplementary Material 1 [file 12885_2026_15748_MOESM1_ESM.docx]

**Clavien-Dindo Classification System**

Grading Criteria:

Grade I: Minor Complications

- No surgical, endoscopic, radiological, or pharmacological intervention required
- Allowed treatments include drugs like antiemetics, antipyretics, analgesics, diuretics, electrolytes, and physiotherapy
- Wound infections at the bedside

Grade II: Complications Requiring Pharmacological Treatment

- Requires pharmacological treatments beyond those allowed in Grade I
- Blood transfusions, total parenteral nutrition
- Medications like antibiotics, anticoagulants

Grade III: Complications Requiring Surgical/Procedural Intervention

- Grade IIIa: Intervention under local anesthesia
- Grade IIIb: Intervention under general anesthesia

Grade IV: Life-Threatening Complications

- Grade IVa: Single organ dysfunction (including dialysis)
- Grade IVb: Multi-organ dysfunction

Grade V: Death

Suffix "d" indicates complications occurring after hospital discharge.

Key Advantages:

1. High objectivity
2. Standardized assessment
3. Facilitates comparative analysis across different studies

Original Citation:
Dindo, D., Demartines, N., & Clavien, P. A. (2004). Classification of surgical complications: a new proposal with evaluation in a cohort of 6336 patients and results of a survey. Annals of surgery, 240(2), 205.
