# Supplementary material for: Efficacy of SLNB in early endometrial adenocarcinoma in China: a retrospective cohort study based on inverse probability of treatment weighting
Source: BMC Cancer. 2026 Feb 25;26:425. doi: 10.1186/s12885-026-15748-2 (PMC13041019; doi:10.1186/s12885-026-15748-2)
Supplement: Supplementary file 2 — Supplementary Material 2 [file 12885_2026_15748_MOESM2_ESM.docx]

**Numeric Rating Scale (NRS) Pain Assessment**

**Patient Information**

- Name: ____________________
- Gender: ____________________
- Age: ____________________
- Department: ____________________
- Ward Number: ____________________
- Hospital Number: ____________________

| **Assessment Parameter** | **Postoperative Day 1** | **Postoperative Day 3** | **Discharge** |
| --- | --- | --- | --- |
| **Time** |  |  |  |
| **Pain Score (0-10)** |  |  |  |
| **Pain Level** |  |  |  |
| **Assessor Signature** |  |  |  |

**Pain Intensity Scale**

**0-10 Numeric Pain Rating Scale**

0 1 2 3 4 5 6 7 8 9 10

|----+----+----+----+----+----+----+----+----+----|

No Pain Moderate Pain Worst Possible Pain

**Detailed Pain Descriptors**

| **Score** | **Pain Intensity** | **Description** | **Clinical Interpretation** |
| --- | --- | --- | --- |
| 0 | No Pain | No discomfort | No intervention required |
| 1-3 | Mild Pain | Slight discomfort | Minimal medical intervention |
| 4-6 | Moderate Pain | Noticeable pain | Active pain management needed |
| 7-9 | Severe Pain | Significant distress | Urgent pain control required |
| 10 | Extreme Pain | Unbearable suffering | Immediate intensive intervention |
