# Supplementary material for: Efficacy of SLNB in early endometrial adenocarcinoma in China: a retrospective cohort study based on inverse probability of treatment weighting
Source: BMC Cancer. 2026 Feb 25;26:425. doi: 10.1186/s12885-026-15748-2 (PMC13041019; doi:10.1186/s12885-026-15748-2)
Supplement: Supplementary file 3 — Supplementary Material 3 [file 12885_2026_15748_MOESM3_ESM.docx]

**Barthel Activities of Daily Living (BADL) Assessment**

**Patient Information**

- Name: ____________________
- Gender: ____________________
- Age: ____________________
- Department: ____________________
- Ward Number: ____________________
- Hospital Number: ____________________

**Assessment Record**

| **Activity** | **Scoring Criteria** | **Time**  **Score** | **Admission** | **6 Hours Post-Surgery Assessment** | **Discharge** |
| --- | --- | --- | --- | --- | --- |
| **Feeding** | Independent eating | 10 |  |  |  |
|  | Needs partial help | 5 |  |  |  |
|  | Completely dependent | 0 |  |  |  |
| **Bathing** | Independent | 5 |  |  |  |
|  | Needs assistance | 0 |  |  |  |
| **Grooming** | Can self-care | 5 |  |  |  |
|  | Needs help | 0 |  |  |  |
| **Dressing** | Independent | 10 |  |  |  |
|  | Partial assistance | 5 |  |  |  |
|  | Completely dependent | 0 |  |  |  |
| **Bowel Control** | Complete control | 10 |  |  |  |
|  | Occasional accidents | 5 |  |  |  |
|  | No control | 0 |  |  |  |
| **Bladder Control** | Complete control | 10 |  |  |  |
|  | Occasional accidents | 5 |  |  |  |
|  | No control | 0 |  |  |  |
| **Toilet Use** | Independent | 10 |  |  |  |
|  | Needs assistance | 5 |  |  |  |
|  | Cannot manage | 0 |  |  |  |
| **Bed to Chair Transfer** | Independent | 15 |  |  |  |
|  | Minimal help | 10 |  |  |  |
|  | Significant assistance | 5 |  |  |  |
|  | Cannot transfer | 0 |  |  |  |
| **Mobility (Walking)** | Independent (50m) | 15 |  |  |  |
|  | Partial assistance | 10 |  |  |  |
|  | Wheelchair use | 5 |  |  |  |
|  | Immobile | 0 |  |  |  |
| **Stair Climbing** | Independent | 10 |  |  |  |
|  | Needs assistance | 5 |  |  |  |
|  | Cannot climb | 0 |  |  |  |
| **Total Score** | | **100** |  |  |  |
| **Dependency Level** | | |  |  |  |
| **Assessor's Signature** | | |  |  |  |

**Dependency Level Interpretation**

- 0-20 points: Total dependence
- 21-40 points: Severe dependence
- 41-60 points: Moderate dependence
- 61-90 points: Mild dependence
- 91-100 points: Independent

**Assessment Notes**

- Score based on actual performance
- Assess most recent functional status
- Involve patient and caregivers in assessment
